# Energy flow differences in throwing arm joints between javelin and weighted balls in male javelin throwers

**DOI:** 10.3389/fspor.2025.1650684

**Published:** 2025-12-10

**Authors:** Hans-Peter Köhler, Kristof Kipp, Nikola Prvulović, Maren Witt

**Affiliations:** 1 Department Biomechanics in Sports, Leipzig University, Leipzig, Germany; 2 Department of Physical Therapy—Program in Exercise Science, Marquette University, Milwaukee, WI, United States; 3 Centre of Research Excellence in Nutrition and Metabolism, Institute for Medical Research, National Institute of Republic of Serbia, University of Belgrade, Belgrade, Serbia

**Keywords:** inverse dynamics, track and field, modeling, optimization, training equipment

## Abstract

**Introduction:**

To enhance release velocity during competition, javelin throwers incorporate implements of varying mass into their training regimens. Previous research has demonstrated that, although velocity contributes quadratically to the computation of kinetic energy, heavier implements generate substantially greater kinetic energy at the moment of release, despite markedly lower release velocities. The primary objective of the present investigation was to analyze energy transfer within the throwing arm to gain deeper insight into the biomechanical mechanisms underlying the use of implements with different masses.

**Methods:**

The three-dimensional coordinates of 16 reflective markers were recorded for 6 athletes during throws using 6 different implement masses, using 12 infrared cameras. Based on this kinematic data, segmental energy transfer was estimated via inverse dynamics using a multi-body modeling approach. Subsequent comparisons were conducted using nonlinear temporal registration and statistical non-parametric mapping.

**Results:**

The results indicate that energy flow at the shoulder joint remains largely consistent across implements of varying mass. However, significant differences in energy transfer were observed at the more distal joints.

**Discussion:**

The findings suggest that the increased kinetic energy observed with heavier implements arises from an internal redistribution of energy within the throwing arm, rather than from greater overall energy input. Consequently, improvements in release velocity associated with lighter or heavier implements are likely attributable to mechanisms other than modifications in energy flow dynamics.

## 1 Introduction

The release speed is the most important target parameter in the javelin throw and is the only release parameter that must be maximized, while all others should be optimized (1, 2). From a mechanical point of view, the athlete must do as much work as possible on the implement to change its kinetic energy and increase its release speed. In contrast to other throwing disciplines, the javelin–athlete system can accumulate large magnitudes of kinetic energy during the run-up. As a result, a substantial portion of the final kinetic energy required to produce high release speeds is already present at the beginning of the delivery phase of the throw. Therefore, the primary objective of the training process is for the athlete to become more technically and physically proficient in transferring kinetic energy from the javelin–athlete system to the implement via the kinematic chain of the torso and upper body. Hence, the shoulder joint plays a decisive role: The greater the energy transferred through the shoulder joint, the higher the velocity that can theoretically be obtained at the instant of release (3–5).

Within the training process of javelin throwers, performing throws with the actual implement is the most specialized form of training. It is also, however, highly physically demanding owing to the large forces and loads experienced by the lower extremities (especially the bracing leg), which often restricts the number of training throws (1). To reduce the mechanical load on the lower extremities, throwers will sometimes perform throws with reduced run-up speeds or use different weighted implements. These adjustments help athletes achieve similar release speeds while maintaining the overall demands on the upper extremity (6).

Several studies on baseball throwing showed that training with both light and heavy throwing implements can improve pitching speed (7). Evidence on the efficacy of different implements for enhancing release speed and javelin performance has been based primarily on experiential accounts (6). Recent work, however, demonstrates that the use of implements of different masses causes changes in kinematics and kinetics. Unlike baseball throwing, in javelin throwing, an increase in mass of the implement leads to an increase in joint moments. Conversely, a reduction in mass produces similar effects on both throwing motions, namely, an increase in throwing speed and joint angular velocities (8, 9).

Moreover, while lighter implements can be thrown with higher velocities, heavier implements require greater work input, even though the velocity contributes quadratically to the equation of kinetic energy (9). The reason why heavy implements require more work is unclear. From a theoretical point of view, there are two possibilities. First, heavier implements may lead to an improvement in the transfer of mechanical energy, thereby transporting more energy through the throwing arm to the implement. Second, due to the lower speed of the implement, the segments of the arm also have lower velocities and therefore lower kinetic energy. This means that more of the energy transferred through the shoulder would be available to accelerate the implement rather than the segments. This aligns line with the statement by Bartonietz (10), who noted that the musculoskeletal system is only capable of producing/ transferring a finite amount of energy per unit time, which therefore limits performance.

Since no studies have investigated upper-body energy flow when throwing implements of different masses, the aim of this study was to investigate how implements of different masses change the energy flow of the throwing arm. In addition, the study sought to determine how energy flow of implements thrown from shorter run-ups differs from that observed in javelin throws from a longer run-up. This would lead to a better understanding of the effects of different implements on energy flow, thereby enabling better planning of their use. This information can be integrated into the training process and used for different purposes such as injury prevention and performance enhancement.

## 2 Methods

### 2.1 Participants

Due to the requirements for throwing different implements and the corresponding necessary expertise, only a limited number of athletes were eligible for this study. Six right-handed male javelin throwers (1.91 ± 0.05 m; 97.08 ± 10.66 kg; personal best: 85.90 ± 9.84 m) participated in the study. All athletes were internationally experienced and familiar with throwing implements of different masses (0.046–3.0 kg). None of the athletes reported injuries at the time of the investigation. Prior to the participation, the athletes were informed about the purpose of the study and provided their consent. The ethics advisory board of Leipzig University approved the study protocol (ethical approval nr: 2021.05.16_eb_98) and the study was conducted in accordance with the Declaration of Helsinki.

### 2.2 Instruments and materials

Each participant was equipped with 16 retroreflective markers (left and right spina iliaca anterior superior, left and right spina iliaca posterior superior, processus xiphoideus, incisura jugularis, 7th cervical vertebrae and 12th thoracic vertebrae, left and right acromion, lateral and medial epicondyle of the humerus, ulnar and radial styloid, metacarpophalangeal joint of the second and fifth fingers). Five markers were attached to the javelin (11), while the balls had no markers.

The three-dimensional positions of the retroreflective markers were captured using 12 infrared cameras (Oqus 7+, Qualisys AB, Gothenburg, Sweden) sampling at 250 Hz and two video cameras for event detection (Oqus 210c, Qualisys AB, Gothenburg, Sweden) operating at 125 Hz. Data were recorded using Qualisys Track Manager (Ver. 2021.1 build 6470). A second camera system consisting of two cameras (Basler ace acA1920–155uc, Basler AG, Ahrensburg, Germany), operating at 100 Hz, was placed on the rear and throw side, with the cameras placed orthogonally to each other (12). This system was used to estimate the release velocity of the implement. The experimental protocol is detailed in the following.

After a warm-up session, each participant performed throws with six different implements. The study was carried out in an indoor facility where all implements were thrown into a net. Five of these were throwing balls (Podium Balls, Birmingham, UK) of the following weights: 1,200, 1,000, 800, 600, and 400 g. In addition, all athletes threw a javelin (GETRA Kinetic 70 m, 800 g, Getrasport, Regensburg, Germany), which was slightly modified for indoor use (4, 13). The order of the implements thrown was randomized. While the javelin was thrown from the longest approach the athletes were capable of at the time point of the investigation, the balls were thrown from a three-step approach (push off from the left leg to the impulse stride—touchdown of the rear leg—touchdown of the bracing leg). Each implement was thrown at least three times. For further analysis, the three throws of each implement with the highest release speed (v_0_) were chosen.

### 2.3 Data processing

The time points of the touchdown of the rear leg, touchdown of the bracing leg, and the release were identified for each trial before further processing. The trajectories of the different markers were filtered using a fourth-order, zero-lag Butterworth filter, with filter frequencies (10–13 Hz) determined by residual analysis (14).

To calculate the kinematics (segment angular velocities and joint linear velocities) and kinetics [resultant joint moments and resultant joint forces (RJF)] needed for energy flow analysis, a five-segment model was built and implemented in Visual 3D (HAS-Motion, Kingston, Ontario, Canada). The model consisted of the thorax, upper arm, forearm, and hand. While the javelin was modeled based on the markers attached to it (4, 11), the balls were modeled as a simple sphere and attached to the center of mass of the hand (15). The joint centers of the wrist and elbow were calculated to be the midpoints between the ulnar and radial styloid processes and the medial and lateral humeral epicondyles, respectively, in accordance with ISB recommendations (16). The shoulder joint was calculated using the functional methods provided by Schwartz and Rozumalski (17) and implemented in Visual 3D (18). Body segment inertial parameters were estimated using the methods provided by de Leva (19). For pose estimation, the six-degrees-of-freedom algorithm was used. Kinetics were calculated as internal RJF and torques (RJT) using the Newton–Euler equations of motion.

After computing kinematics and kinetics for the segments (hand, forearm, upper arm) and joints (wrist, elbow, shoulder joint) of the throwing arm, a custom written script in MATLAB (Ver. 23.2.0; The Mathworks Inc., Natick, MA, USA) was used to conduct a segmental power analysis where the rates at which energy leaves or enters a segment via the RJT or RJF were calculated according to Robertson and Winter (20). Specifically, the time series of the joint force power (JFP), defined as the power delivered by the RJF, was calculated as

$$\mathrm{JFP}=F_{i,j}\cdot \;v_{j},$$

and the time series of the segment torque power (STP) was computed as

$$\mathrm{STP}=T_{i,j}\cdot \;{\dot{\theta }}_{i,j}.$$

Here, $F_{i,j}$ and $T_{i,j}$ denote the RJF and RJT, respectively, of the *i*-th segment of the *j*-th joint. $v_{j}$ and ${\dot{\theta }}_{i,j}$ denote the linear velocity of the *j*-th joint and the angular velocity of the *i*-th segment at the *j*-th joint, respectively. While $F_{i,j}$ and $T_{i,j}$ are equal in magnitude but opposite in direction for the two segments connected by a joint, $v_{j}$ is equal for both segments. The ${\dot{\theta }}_{i,j}$, on the other hand, can vary between the two segments of a joint. As a result, while the JFP represents the rate of energy loss of one segment and the rate of energy gain of the other, the STP represents not only the rate of transfer of mechanical energy but also the rate of energy generation and absorption at the considered joint as outlined in Table 1 (5, 20, 21). To calculate net energy transfer (TP) of each joint from proximal to distal, the sum of JFP and the transfer component of STP of the distal segment were computed (5).

**Table 1:** Calculation of the transfer, generation, and absorption of mechanical energy depending on magnitude and sign of the STP of both segments of a joint (4, 5, 20).

	Generation	Absorption	Transfer
Same sign			
Both positive	To proximal segment at T_p_θ_p_ to distal segment at T_d_θ_d_	0	0
Both negative	0	From proximal segment at T_p_θ_p_ from distal segment at T_d_θ_d_	0
Opposite sign			
|STP_p_| > |STP_d_|			
+ −	To proximal segment at T_p_(θ_p_ − θ_d_)		To proximal segment at T_d_θ_d_
− +	0	From proximal segment at Tp(θ_p_ − θ_d_)	To distal segment at T_d_θ_d_
|STP_p_| < |STP_d_|			
+ −		From distal segment at T_d_(θ_d_ − θ_p_)	To proximal segment at T_p_θ_p_
− +	To distal segment at Td(θ_d_ − θ_p_)	0	To distal segment at T_p_θ_p_

STP_p_, segment torque power of the proximal segment; STP_d_, segment torque power of the distal segment; T_p_, proximal joint torque; T_d_, distal joint torque; θ_p_, angular velocity of the proximal segment; θ_d_, angular velocity of the distal segment.

Subsequently, the different power–time series were integrated over time from the touchdown of the rear leg to the release to account for the energy transferred, generated, absorbed, and moved by the JFP and STP (4).

### 2.4 Statistics

To compare the different power–time series among the different implements, the MATLAB package for statistical non-parametric mapping (SnPM1d, Ver. M.0.4.10) was utilized (22–24). The power–time series were first time-normalized to 101 data points from the touchdown of the rear leg to the instant of release. To better distinguish between amplitude and temporal effects, the different power continua were also processed in a non-linear using manner the wrapper functions provided by Pataky et al. (25) in Python 3.8.11 (26). This procedure yields two different continua for each curve, where the first one contains the displaced power–time series; moreover, the different indices of each curve are moved such that certain events like local optima are time matched (“warped”) due to which the data points are not equidistant. This type of curve can be used to determine amplitude effects. The second curve is called the displacement field (25). This curve yields information about how much a certain point must be moved to match the same event (e.g., maximum value) in other curves. The greater the amplitude, the greater the shift, whereby the sign determines whether the shift is forward (positive) or backward (negative).

The two different curves were subsequently analyzed across the different implements using the non-parametric version of a one-factorial analysis of variance with repeated measures (ANOVArm) implemented into SnPM1d. Bonferroni-corrected *post-hoc* comparisons were conducted when the omnibus test revealed significant differences among the implements. Friedman's Test was used to compare the energy transferred, generated, and absorbed, with Conover's Test used for *post-hoc* comparison. A type I error probability of *α* = 5% was adopted for both the omnibus and *post-hoc* tests. The *p*-values of the *post-hoc* comparisons were corrected using the Bonferroni–Holms method. As a measure of effect size Kendall's W was calculated for the omnibus test and Hedges’ g for *post-hoc* comparisons. The effect sizes were interpreted using Cohen’s (27) guidelines, where values of 0.1–<0.3 signify a small effect, 0.3–<0.5 signify a medium effect, and ≥0.5 signify a large effect. For all statistical tests, the type I probability was set to *α* = 0.05.

## 3 Results

**Figure 1:**
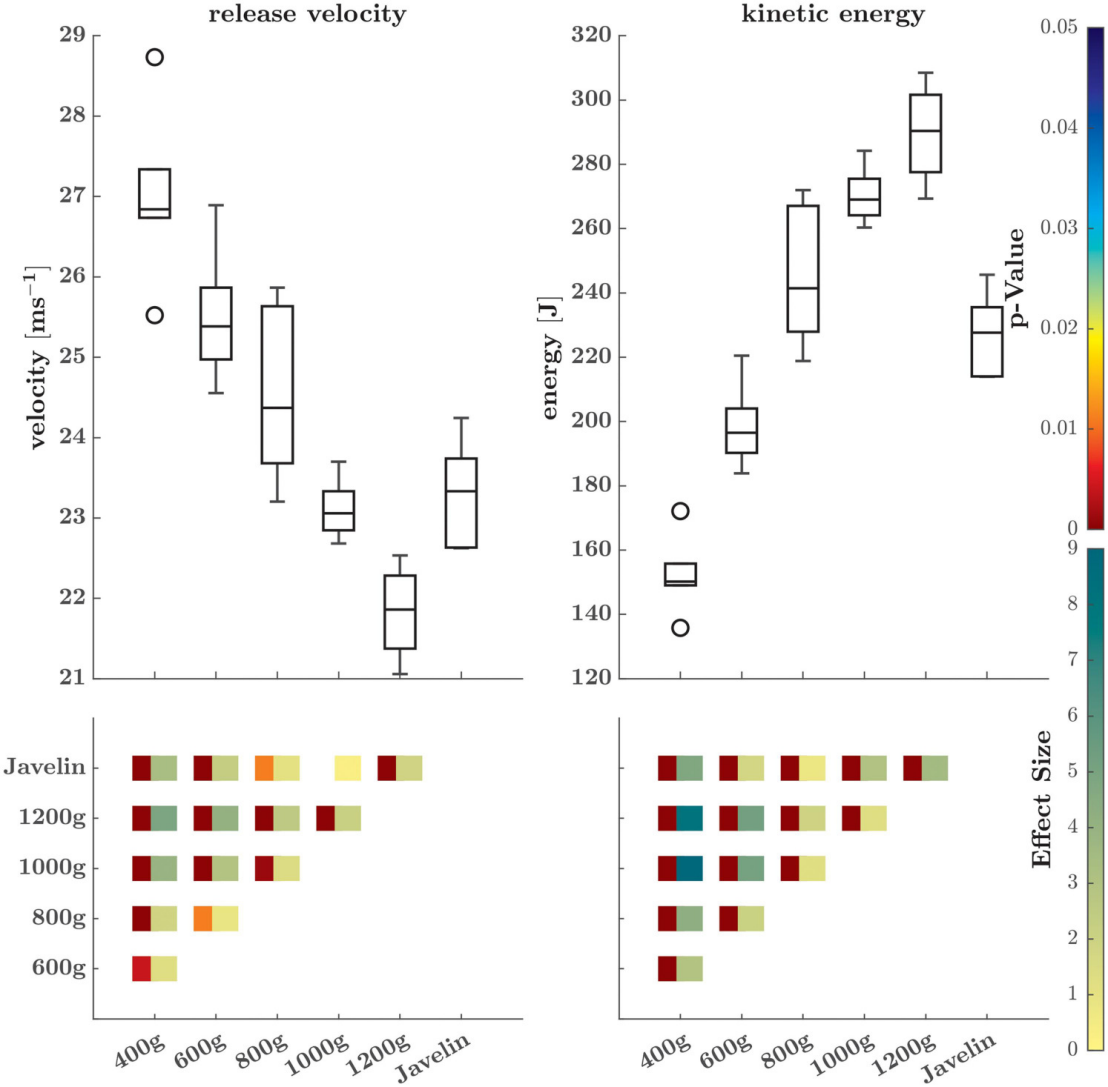
Release velocity (left) and kinetic energy (right) of the different implements at release. The second row marks the *p*-value (left squares, only if there are significant differences) and effect size (right squares) matrix of the *post-hoc* comparisons. The meaning of the colors is indicated on the right side.

The release velocity (*Χ*^2^ = 27.14; *p* < 0.001; W = 0.905) and the subsequently calculated kinetic energy (*Χ*^2^ = 28.95; *p* < 0.001; W = 0.965) both showed significant main model effects. In particular, the release velocity increased as implement weight decreased, whereas the kinetic energy decreased as the implement weight decreased (Figure 1).

**Figure 2:**
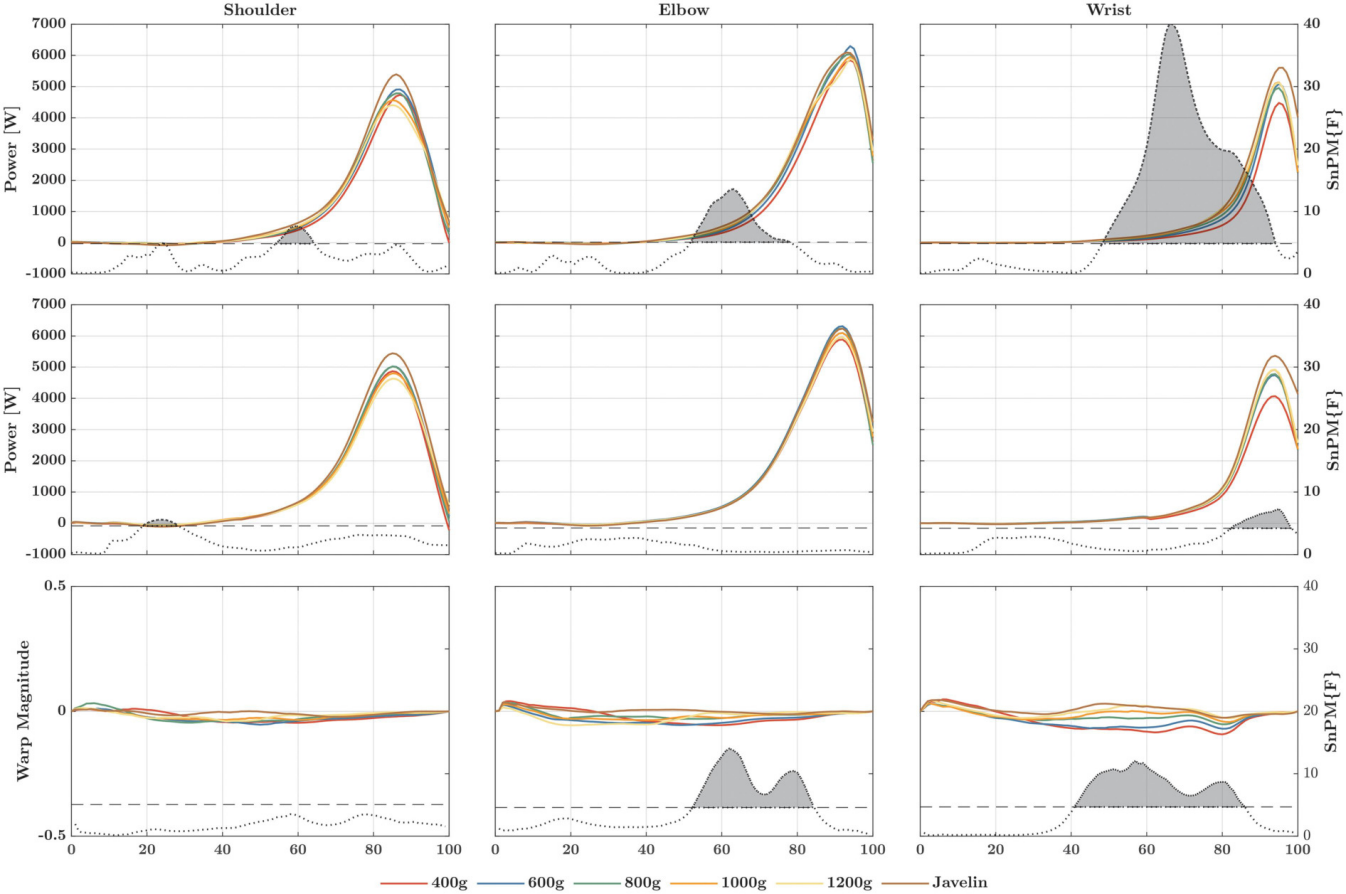
Linear registered (first row) and non-linear registered power–time curves (second row) of the rate at which energy is transferred to the distal segment at the shoulder (left column), elbow (middle column), and wrist (right column). The third row marks the warp magnitude, i.e., how much the corresponding point in the power–time curve must be moved in time to be in this place to match the same point of the other points (e.g., the maximum value). The higher the amplitude, the greater the shift, whereby the sign determines whether the shift is forward (positive) or backward (negative). In each graph, the dotted curve represents the F-value at the corresponding point in time. If this curve exceeds the critical threshold (dashed line), there is a significant main effect, also indicated by a gray area beneath the curve. The post-hoc comparisons can be found in Supplementary Data Sheet 1.

**Figure 3:**
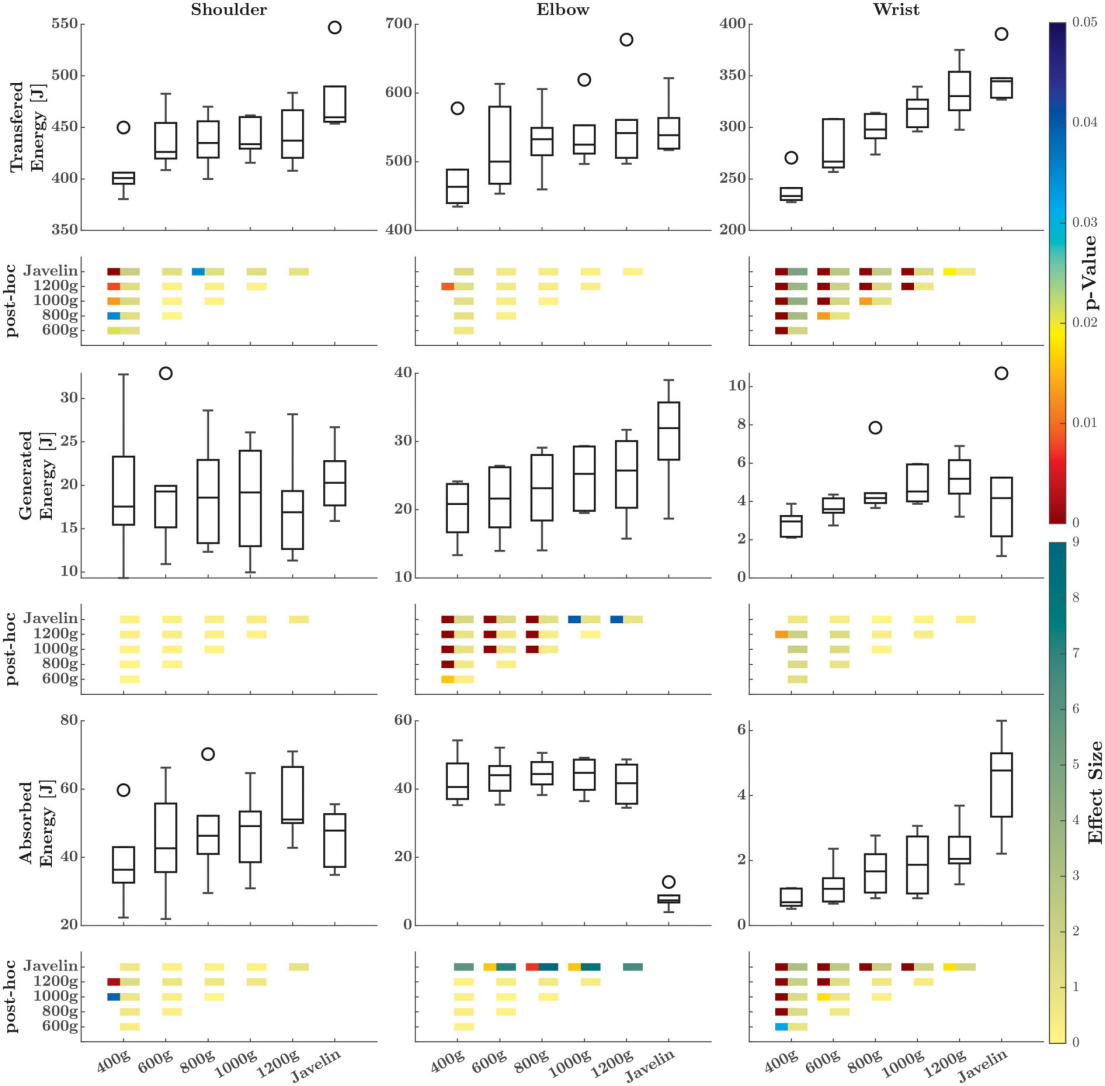
Boxplots of the energy transferred (first row), generated (third row), and absorbed (sixth row) to the distal segment at the shoulder (first column), elbow (second column), and wrist (third column) for the different implements. The comparison matrix beneath each boxplot contains the *p*-values (left squares, only if there are significant differences) and effect sizes (right squares) of the *post-hoc* comparisons. The meaning of the colors is indicated on the right side.

For the rate at which energy is transferred distally (Figure 2), SnPM1d revealed both temporal and magnitude effects across the different implements and joints. At the shoulder, *post-hoc* analysis revealed only magnitude effects, whereas at the elbow joint, it revealed only temporal effects. In this case, the rise of the power–time curve was delayed at approximately 50% as implements weight decreased. At the wrist joint, both magnitude and temporal effects were present. Specifically, the 400-g ball and the javelin displayed different magnitude effects compared to the other implements. The temporal effects were similar to those reported for the elbow joint, in that the lighter implements led to a delayed rise of the power–time curve at approximately 80% of the cycle. In addition, the energy transferred differed significantly among all implements (Figure 3) at the shoulder (*Χ*^2^ = 19.05; *p* = 0.002; W = 0.635), elbow (*Χ*^2^ = 13.14; *p* = 0.022; W = 0.438), and wrist joints (*Χ*^2^ = 28.48; *p* < 0.001; W = 0.949). At each of the three joints, more energy was transferred when the athletes threw the heavier implements. The influence of this effect became stronger (measured by the effect sizes) as more distal joints were analyzed.

**Figure 4:**
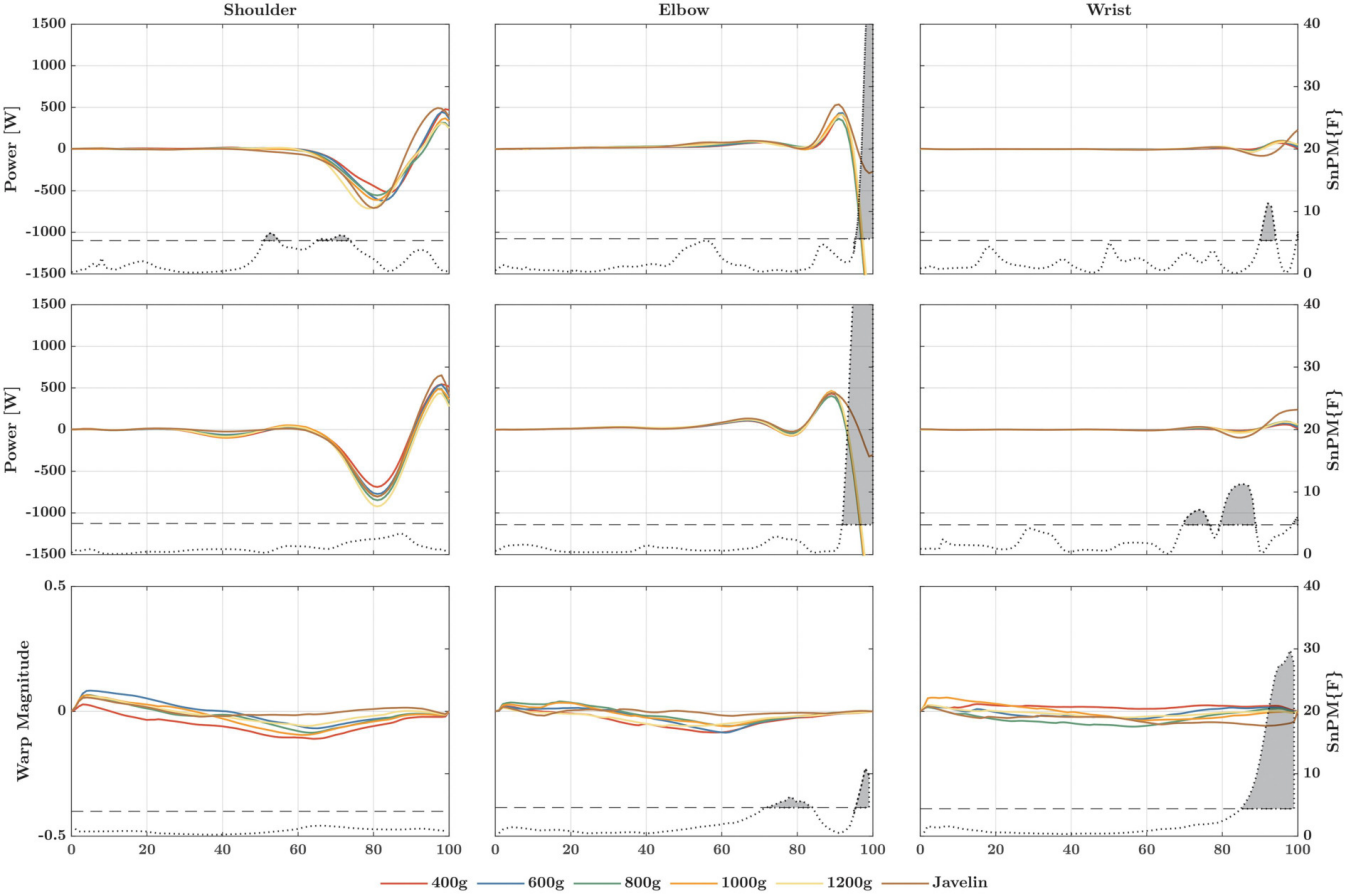
Linear registered (first row) and non-linear registered power–time curves (second row) of the rate at which energy is generated (positive values) and absorbed (negative values) to the distal segment at the shoulder (left column), elbow (middle column), and wrist (right column). The third row marks the warp magnitude, i.e., how much the corresponding point in the power–time curve must be moved in time to be in this place to match the same point of the other points (e.g., the maximum value). The higher the amplitude, the greater the shift, whereby the sign determines whether the shift is forward (positive) or backward (negative). In each graph, the dotted curve represents the F-value at the corresponding point in time. If this curve exceeds the critical threshold (dashed line), there is a significant main effect, also indicated by a gray area beneath the curve. The post-hoc comparisons can be found in Supplementary Data Sheet 2.

For the rate at which energy was generated or absorbed (Figure 4), SnPM1d analysis revealed temporal and magnitude effects only at the elbow and wrist joints. At the elbow, these effects only occurred for the comparison between the javelin and the different ball weights. At the wrist, there were additional magnitude effects for the lightest ball compared to heavier implements. At the shoulder joint (Figure 3), no differences were found among the implements (*Χ*^2^ = 2.0; *p* = 0.849; W = 0.067), whereas the absorbed energy did differ among the implements (*Χ*^2^ = 14.38; *p* = 0.013; W = 0.479), with greater energy absorbed as the weight of the implements increased. Conversely, both the energy generated (*Χ*^2^ = 27.24; *p* < 0.001; W = 0.908) and absorbed (*Χ*^2^ = 14.00; *p* = 0.016; W = 0.467) at the elbow joint differed significantly. The generation of energy increased as the weight of the implements increased, with the only difference being energy absorption between the balls and the javelin. The same trend also held for the wrist joint where the energy generated (*Χ*^2^ = 13.24; *p* = 0.021; W = 0.441) and absorbed (*Χ*^2^ = 25.71; *p* < 0.001; W = 0.857) significantly differed, with slightly greater energy generation and absorption at greater implement weights.

**Figure 5:**
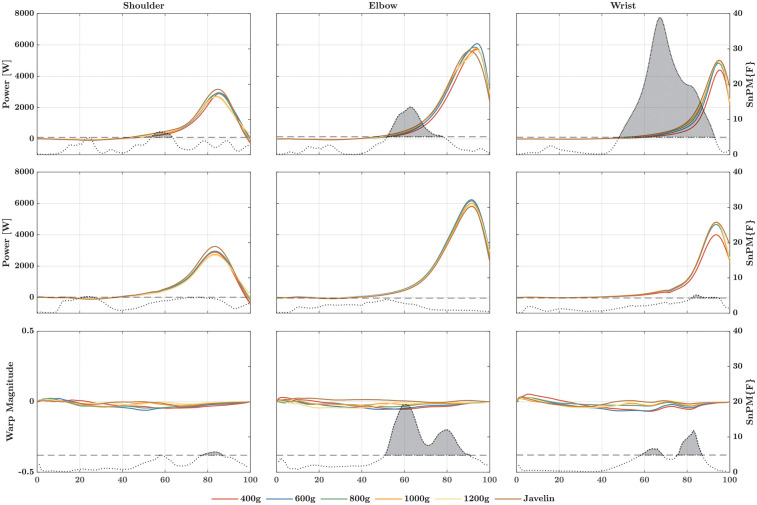
Linear registered (first row) and non-linear registered power–time curves (second row) of the rate at which energy flows to the distal segment due to the joint forces at the shoulder (left column), elbow (middle column), and wrist (right column). The third row marks the warp magnitude, i.e., how much the corresponding point in the power–time curve must be moved in time to be in this place to match the same point of the other points (e.g., the maximum value). The higher the amplitude, the greater the shift, whereby the sign determines whether the shift is forward (positive) or backward (negative). In each graph, the dotted curve represents the F-value at the corresponding point in time. If this curve exceeds the critical threshold (dashed line), there is a significant main effect, also indicated by a gray area beneath the curve. The post-hoc comparisons can be found in Supplementary Data Sheet 3.

**Figure 6:**
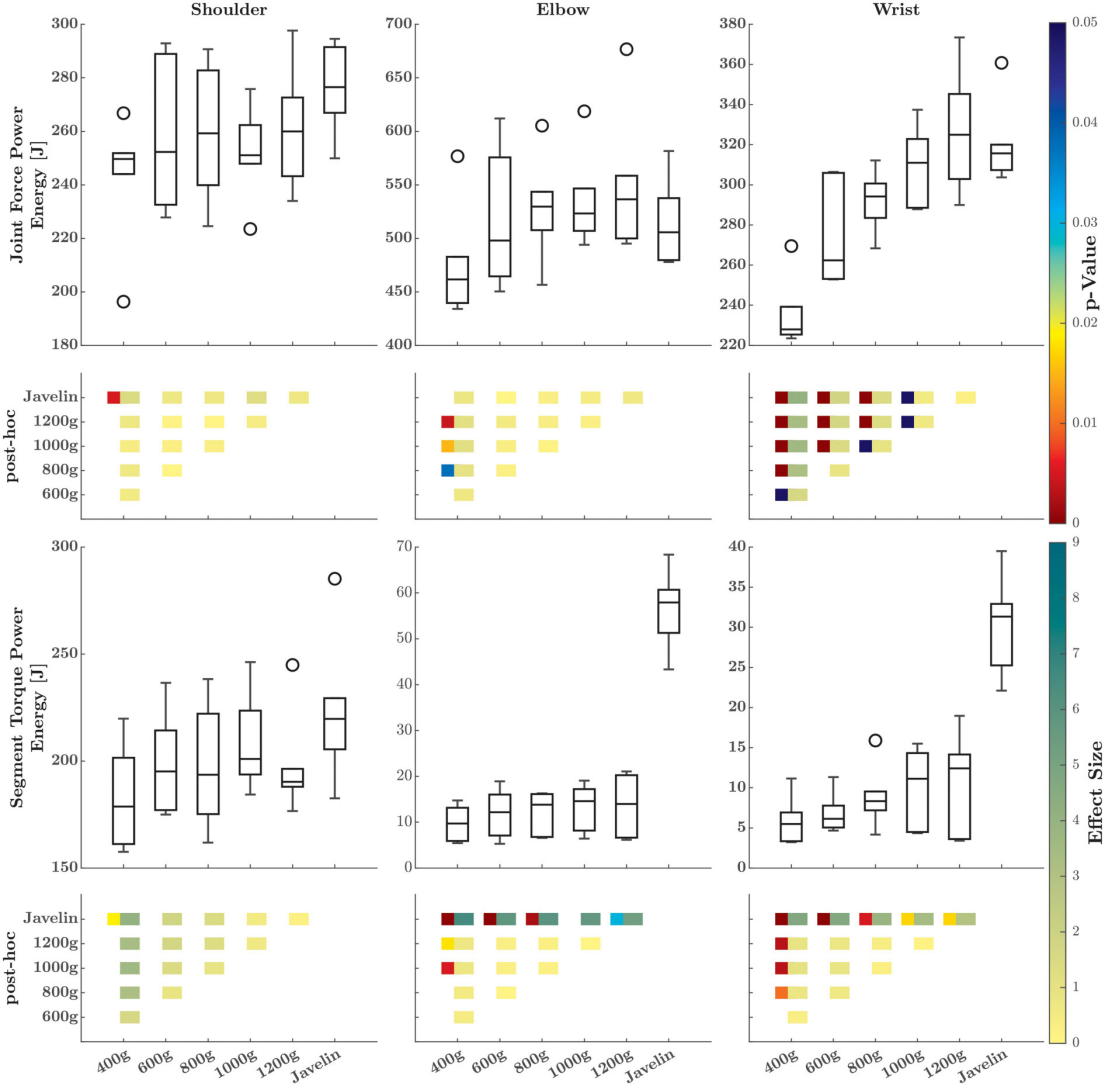
Boxplots of the energy flowing to the distal segment due to the joint forces (first row) and the segment torques (third row) at the shoulder (first column), elbow (second column), and wrist (third column) for the different implements. The comparison matrix beneath each boxplot contains the *p*-values (left squares, only if there are significant differences) and effect sizes (right squares) of the *post-hoc* comparisons. The meaning of the colors is indicated on the right side.

For the JFP (Figure 5), the SnPM1d analysis of the power–time curves revealed significant temporal and magnitude effects for all joints. At the elbow, no magnitude effects were found; however, *post-hoc* comparisons revealed differences for the javelin at the shoulder and for the 400-g implement. At all three joints, several temporal effects were present with the lighter implements, indicating a delay in the rise of the power–time continua. For the energy transferred by the JFP in a proximal-to-distal manner (Figure 6), the Friedman test revealed significant differences at the shoulder (*Χ*^2^ = 13.14; *p* = 0.022; W = 0.438), elbow (*Χ*^2^ = 14.19; *p* = 0.014; W = 0.473), and wrist joints (*Χ*^2^ = 26.48; *p* < 0.001; W = 0.883). At the shoulder, differences were present only between the 400-g ball and the javelin, while the elbow and wrist showed additional differences, with greater energy transferred by the JFP when athletes threw heavier implements.

**Figure 7:**
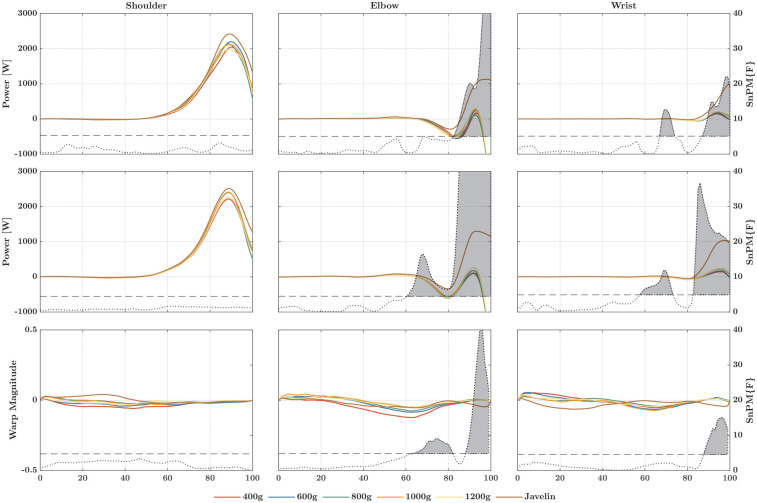
Linear registered (first row) and non-linear registered power–time curves (second row) of the rate at which energy flows to the distal segment due to the segment torques at the shoulder (left column), elbow (middle column), and wrist (right column). The third row marks the warp magnitude, i.e., how much the corresponding point in the power–time curve must be moved in time to be in this place to match the same point of the other points (e.g., the maximum value). The higher the amplitude, the greater the shift, whereby the sign determines whether the shift is forward (positive) or backward (negative). In each graph, the dotted curve represents the F-value at the corresponding point in time. If this curve exceeds the critical threshold (dashed line), there is a significant main effect, also indicated by a gray area beneath the curve. The post-hoc comparisons can be found in Supplementary Data Sheet 4.

The STP at the proximal end of the distal segment of a joint (Figure 7) showed magnitude and amplitude effects among the different implements only at the elbow and wrist joints. While the javelin produced magnitude effects for the elbow and wrist joints in comparison to the other implements, *post-hoc* pairwise comparisons at the elbow revealed only temporal effects. The energy induced distally by the STP (Figure 6) showed significant differences for the shoulder (*Χ*^2^ = 11.91; *p* = 0.036; W = 0.397), elbow (*Χ*^2^ = 20.67; *p* < 0.001; W = 0.689), and wrist joints (*Χ*^2^ = 21.62; *p* < 0.001; W = 0.721). At the wrist and the elbow, the javelin showed significantly higher values compared to the other implements, while at the wrist, only the 400-g ball was different from the other implements.

## 4 Discussion

The aim of this study was to investigate the effects of different implements on energy flow at the joints of the throwing arm. To our knowledge, this is the first study to examine the effects of different implements on energy flow in any throwing-related sport. The results show that only minor changes occur in the various performance and energy terms at the shoulder. In contrast, clear temporal and magnitude effects were observed at the elbow and wrist joints.

As the implement mass increased, the release speed decreased, which is consistent with findings in baseball and handball (8, 28). Interestingly, however, this relationship is reversed when considering kinetic energy at release. Specifically, throwing heavier implements required more work to be done on the implement, even though lighter implements achieved higher release speeds. This finding is noteworthy because speed is squared within the kinetic energy calculation, which implies that the selecting the appropriate implement weight is important to balance the velocity and work conditions. In this context, it should be noted that the javelin was thrown from a longer/faster run-up and lower throwing speeds were achieved in comparison to some of the lighter implements. The release speed and kinetic energy obtained with the competition implement were similar to those achieved with the 800–1,000-g ball.

At the joint level, the energy delivered to the distal segment through energy transfer showed larger differences among the balls as more distal joints were analyzed. At the wrist, the increase in energy was primarily due to the JFP delivered to the joint, which depended on temporal effects rather than magnitude effects. In other words, throwing heavier implements initiated mechanical energy transfer by the joint forces that subsequently resulted in greater distal energy transfer.

As ball weight increased, energy both absorbed and generated at the wrist also increased, while at the elbow, only the generated energy increased. Owing to the lower speeds of the heavier implements, it can be hypothesized that the working conditions for the muscles change, thereby enabling the muscles to perform more work. However, compared to the javelin, the active contribution at the elbow was significantly lower for the balls. This may be due to the javelin's shape and greater moments of inertia, which provide more resistance so the muscles at the elbow can perform better (9). Furthermore, optimal acceleration of the javelin requires that the release velocity vector and the longitudinal axis of the implement are as closely aligned as possible. It is plausible that better control of the javelin (i.e., alignment of the velocity vector and implement axis) is disrupted by excessive fluctuation in energy absorption and generation at the wrist, which would explain some of the joint-specific differences in energetics between throwing of the javelin and the other implements.

When throwing the javelin, less energy is absorbed at the elbow than when throwing the other implements. While this may be attributed to differences in the shape of the javelin and the resulting change in hand position, as discussed earlier, it is also important to consider the timing of when these differences occur. For example, most of the energy at the elbow is absorbed by joint torque powers when the elbow is extended rapidly at the end of the delivery phase (>90%). While there are no significant differences in the angular velocities of elbow extension (9), there are significant differences in the joint torque powers at the elbow. It can therefore be concluded that there is a difference between balls and javelins in regard to both the rotational and linear kinetic components, especially at elbow and wrist, which may indicate different “active” contributions from muscles and thus different strategies for accelerating the respective implements. That said, it remains questionable whether implements of different shapes and masses (i.e., balls) can replicate the joint energetics of the distal segments (i.e., the elbow and wrist) observed during throwing of the javelin, particularly when taking the rotational kinetic component into account.

At the shoulder, neither the power terms nor the calculated energies funneled by these terms differed between the implements and javelin, in contrast to the findings for the two distal joints. It can therefore be concluded that power generation at the shoulder and the transfer of kinetic energy to the throwing arm via the shoulder are broadly limited, which may thus represent a general limiting factor in throwing performance (10). However, this finding has to be investigated further to better understand the limiting mechanisms of the shoulder.

While the flow of energy cannot be trained through the use of implements of different weights, training programs with these devices can nevertheless lead to an increase in release velocity (7). This increase must therefore be attributed to other factors, such as an increase in joint moments (with heavier implements) or angular velocities (with lighter implements) (8, 9). Since no additional energy can be transferred through the shoulder to the throwing arm, it must be assumed that the extra energy delivered to heavier implements is freed through a redistribution of energies within the throwing arm. The slower release speeds observed with the heavier implements likely require slower (angular) velocities of the upper arm and lower arm segment, thereby freeing additional energy (8, 9, 28). This redistribution of energy may be used to accelerate the implement and enable greater transfer of energy through the distal joints, as demonstrated by the results.

## 5 Limitations

The results presented must be interpreted in light of some limitations. First, the study was carried out using a small sample size, which limits the statistical tests and their power. Given the examined mean effect size of 0.613 for the omnibus test of the scalar values, the type I error probability of *α* = 5, and the sample size of 6, the actual power was 0.173 (or the type II error probability is *β* = 0.827). To reach an acceptable type II error probability of *β* = 0.10 for the given mean effect size, 36 athletes would be needed. The athletes included in this study, however, were highly experienced and familiar with different types of throwing implements. The high experience level limited the recruitment pool, and expanding the sample to include inexperienced athletes could have led to changes in the results. Second, the study was completed a few months prior to the competition season. Therefore, the athletes were not yet at their peak performance capacity. Third, since the experiment was conducted indoors, the environment did not represent the current javelin throwing conditions. In addition, the indoor experiment resulted in the usage of release speed as a performance parameter rather than throwing distance. Results may not be transferable without limitations because competition performance is heavily influenced by release speed but not exclusively. Moreover, motion capture and multi-body modeling have several limitations. Errors can occur in the movement of the markers, in the estimation of the inertia parameters of the body segments, and in the calculation of the joint centers. However, the procedures outlined in the methods were chosen to minimize these influences within the framework of the study.

## 6 Conclusion and perspectives

Considering only the energetics of the implements when making decisions about practice design may be misleading. Although heavier implements display greater kinetic energy at release, the energy entering the throwing arm due to transfer or generation of mechanical energy does not change. The mechanism underlying the greater energy of the heavier implements at release appears to be the result of lower speeds of the segments, which free energy that can subsequently be transferred to the implements. Therefore, the shoulder joint plays a key role in achieving high release speeds: The greater the energy passed through, the higher the release speed. This further indicates that mechanical performance also seems to be a limiting factor in throwing performance (10). Accordingly, athletes must prepare their shoulder to increase the energy entering the throwing arm, specifically by changing the joint torques and angular velocities at the shoulder to reach higher release speeds (4). This may be done by employing a more rotational style of throwing (29).

Furthermore, while different implements cannot alter energy flow, they can be used to alter specific aspects of mechanical energy, namely, force/torque or angular velocities (9). These may lead to improvements in energy flow by working on both sides of the force–velocity curve (30). Therefore, different implement weights can be used to create an individual force–velocity profile, thereby providing better training recommendations for the following training period. However, further research is required to discover the mechanisms by which training with heavy and light implements leads to improvements in release speeds.

From a practical perspective, only a mixture of implements of different masses can influence the energetics of the shoulder due to changes in strength and speed abilities. A mixture of light and heavy implements should therefore be used to prepare the shoulders for improved energy transfer. However, it is also necessary to determine the extent to which the approach speed influences changes in energy transfer.

## Data Availability

The raw data supporting the conclusions of this article will be made available by the authors, without undue reservation.
