# Supplementary figures and images for: Energy flow differences in throwing arm joints between javelin and weighted balls in male javelin throwers
Source: Front Sports Act Living. 2025 Dec 10;7:1650684. doi: 10.3389/fspor.2025.1650684 (PMC12730163; doi:10.3389/fspor.2025.1650684)

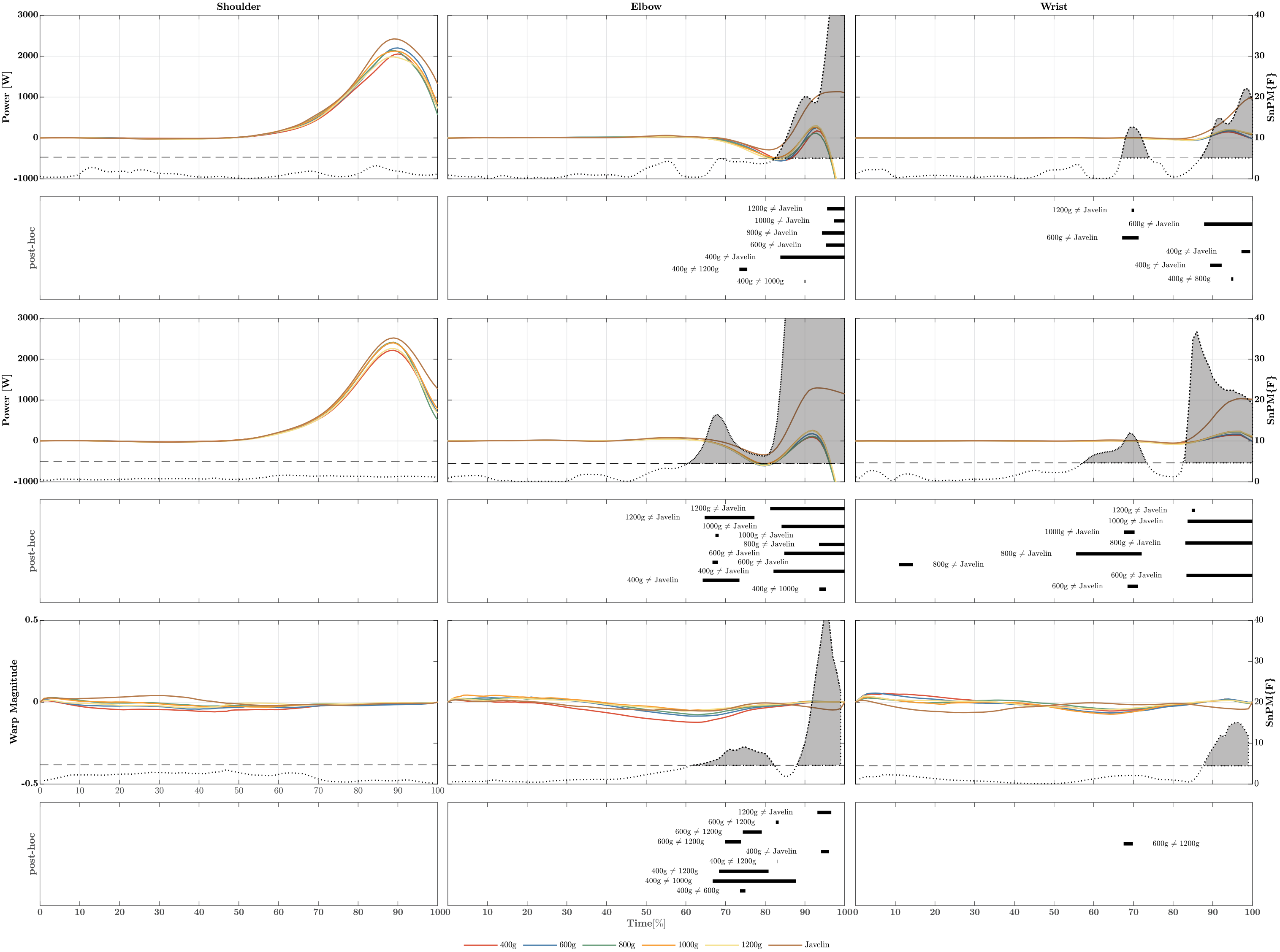

Supplement: Supplementary file 1 [file Datasheet1.pdf]

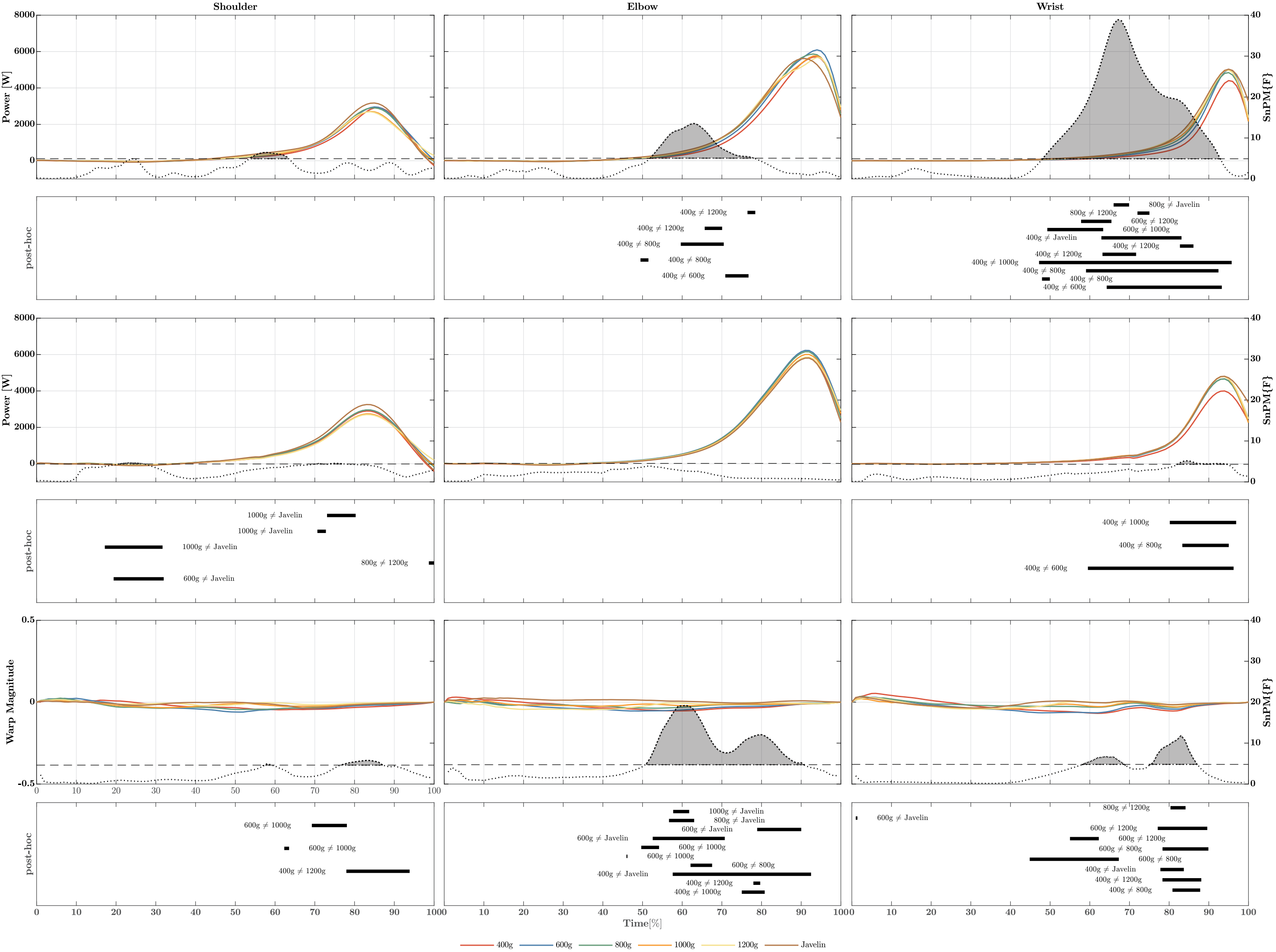

Supplement: Supplementary file 2 [file Datasheet2.pdf]

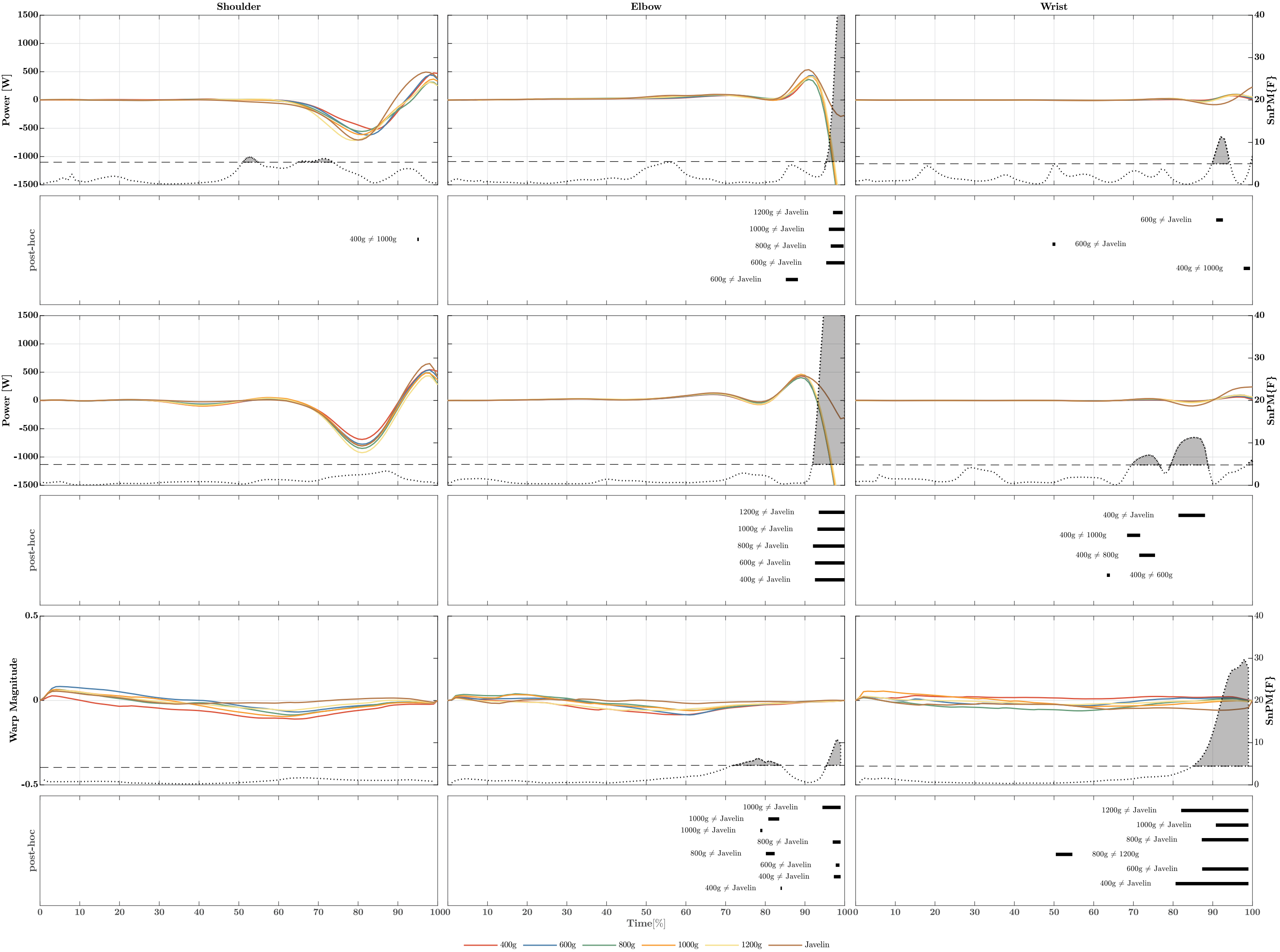

Supplement: Supplementary file 3 [file Datasheet3.pdf]

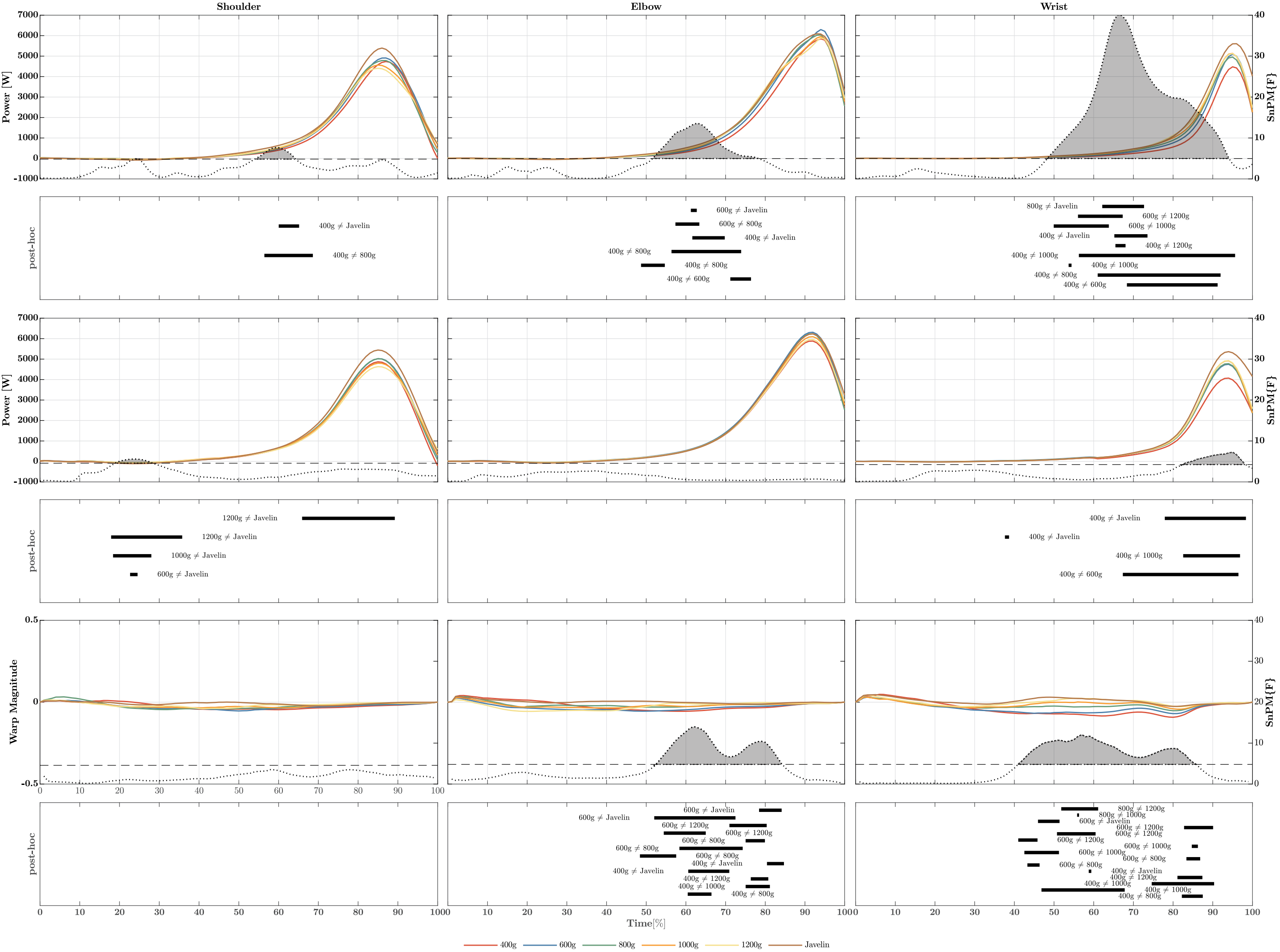

Supplement: Supplementary file 4 [file Datasheet4.pdf]
